# Appearance before performance? Nutritional constraints on life‐history traits, but not warning signal expression in aposematic moths

**DOI:** 10.1111/1365-2656.13103

**Published:** 2019-10-23

**Authors:** Carita Lindstedt, Kaisa Suisto, Johanna Mappes

**Affiliations:** ^1^ Department of Biological and Environmental Sciences University of Jyväskylä Jyvaskyla Finland

**Keywords:** diet, genotype‐by‐environment interaction, melanin, phenotypic plasticity, signal evolution

## Abstract

1. Trade‐offs have been shown to play an important role in the divergence of mating strategies and sexual ornamentation, but their importance in explaining warning signal diversity has received less attention. In aposematic organisms, allocation costs of producing the conspicuous warning signal pigmentation under nutritional stress could potentially trade‐off with life‐history traits and maintain variation in warning coloration.
2. We studied this with an aposematic herbivore *Arctia plantaginis* (Arctiidae), whose larvae and adults show extensive variation in aposematic coloration. In larvae, less melanic coloration (i.e. larger orange patterns) produces a more efficient warning signal against predators, whereas high amounts of melanism (smaller orange pattern) enhance thermoregulation, correlate with better immunity and make individuals harder to detect for naïve predators.
3. We conducted a factorial rearing experiment with larvae originating from lines selected for either small or large orange signal size, which were reared on an artificial diet that had either low or high protein content. Protein content of the diet is critical for melanin production. We measured the effects of diet on individual coloration, life‐history traits, immune defence and reproductive output. We also compared the responses to dietary conditions between the small and large larval signal genotypes.
4. Protein content of the diet did not affect warning coloration in the larval stage, but larval signal sizes differed significantly among selection lines, confirming that its variation is mainly genetically determined. In adults, signal line or diet did not affect coloration in hindwings, but males' forewings had more melanin on the high than on low protein diet. Contrary to coloration, diet quality had a stronger impact on life‐history traits: individuals developed for longer had smaller hindwing sizes in females and lower immune defence on the low protein content diet compared with the high. These costs were higher for more melanic larval signal genotypes in terms of development time and female hindwing size.
5. We conclude that low plasticity in warning signal characteristics makes signal expression robust under varying dietary conditions. Therefore, variation in diet quality is not likely to constrain signal expression, but can have a bigger impact on performance.

## Graphical abstract

Under limited protein resources for the pigment synthesis, aposematic wood tiger moths (*Arctia plantaginis*) prioritize investment in their defensive colour pattern in comparison with performance and size‐associated traits in both larval and adult stages. This low plasticity in warning signal characteristics makes signal expression robust under varying dietary conditions.

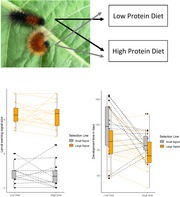

## 1 INTRODUCTION

Predation is one of the most important selection pressures that shapes morphological, behavioural and life‐history strategies in organisms. One way to protect from predation risk is via aposematic displays. Aposematic animals often use conspicuous pigmentation, such as red, orange, yellow and white combined with black pattern elements to advertise their unprofitability to potential predators (Ruxton, Sherratt, & Speed, 2004). In principle, selection by predators is expected to decrease variation in aposematic coloration (Beatty, Beirinckx, & Sherratt, 2004; Joron & Mallet, 1998; Kapan, 2001; Mallet & Barton, 1989; Müller, 1879). It should also favour larger sizes of bright pattern elements (Forsman & Merilaita, 1999; Lindstedt, Lindström, & Mappes, 2008; Lindström, Alatalo, Mappes, Riipi, & Vertainen, 1999) as both of these characteristics combined increase the avoidance learning efficacy of predators. Therefore, predation should favour the most conspicuous and locally abundant signals as it ensures that message gets effectively through to the predators (Arias et al., 2016). This is evident from examples such as wing pattern mimicry in butterflies where selection by predators has resulted in unrelated species developing similar wing patterns to avoid attacks by predators (Kapan, 2001; Müller, 1879).

To determine what maintains intraspecific diversity in warning coloration, the majority of research has (understandably) focused on natural and sexual selection. Much less work has studied warning signal evolution from the life‐history point of view (Mappes, Marples, & Endler, 2005). However, trade‐offs among different traits have been shown to play a critical role in broader patterns of evolutionary diversification at both micro‐ and macro‐evolutionary scales in many sexually selected traits (Simmons, Lüpold, & Fitzpatrick, 2017). Therefore, to estimate the importance of life‐history trade‐offs in warning signal evolution and divergence, we need to determine how environmental conditions shape the life‐history evolution of aposematic organisms and combine this knowledge with information on how different signal patterns are selected for in nature.

In general, how organisms allocate resources among different traits under limited resources is likely to be hierarchical and this hierarchy genetically determined. According to the allocation‐tree model by de Jong (1993), after acquisition of a resource, the first major allocation (e.g. allocation between the growth and somatic maintenance) is most likely to result in a negative covariance between traits. Instead, allocations higher in the allocation tree are more likely to lead to positive covariance between traits (e.g. growth and reproductive traits). Traits in a trade‐off tree will therefore show clusters, with traits showing positive correlations within the cluster and negative correlations between different clusters. Furthermore, the structures prioritized for investment could be those that then return the greatest fitness (Emlen & Nijhout, 2000). What follows is that if we quantify only a few traits that all belong to one of these clusters higher in the allocation tree, we may fail to expose the trade‐offs happening lower in the tree that in turn can shape the evolution of traits higher in a trade‐off tree. Similarly, if we quantify allocation costs experimentally and measure only one or two traits of interest, we can miss critical information of the costs if that trait happens to be the one that organism prioritizes for investment. Finally, in organisms that go through several different life stages such as insects, we need to consider resource allocations across life stages in addition to allocations within life stages to expose trade‐offs critical for their fitness (Burdfield‐Steel, Brain, Rojas, & Mappes, 2018; Lindstedt, Schroderus, Lindström, Mappes, & Mappes, 2016).

Here, we considered these different aspects outlined above by examining how nutritional conditions constrain warning colour expression directly and via trade‐offs in resource allocation between signal and life‐history traits. Organisms need to acquire the pigments deposited to form warning signal patterns directly from their diet (e.g. carotenoids) or, alternatively, synthesize them de novo based on precursors and energy derived from the food (e.g. melanin compounds). Therefore, the quality and availability of a suitable diet forms one potentially important selection pressure on warning signal pigmentation. For example, melanin pigments are nitrogen‐rich large polymers that animals need to synthesize themselves (Sugumaran, 2002). Recent studies have shown that production of melanin pigmentation can be sensitive to certain dietary conditions, such as availability of protein in the diet, and increase diet‐mediated variation in cryptic coloration (Lee, Simpson, & Wilson, 2008). These limitations can be especially critical for herbivorous insects, due to the low levels of usable nitrogen in foliage (Morehouse & Rutowski, 2010; Ojala, Lindström, & Mappes, 2007; Safranek & Riddiford, 1975; Talloen, Van Dyck, & Lens, 2004; Windig, 1999). As a result, these same amino acid precursors are likely to compete with other important functions such as the formation of chitin, cocoons and immunological defence (Andersen, 1979; Lee et al., 2008).

**Figure 1:**
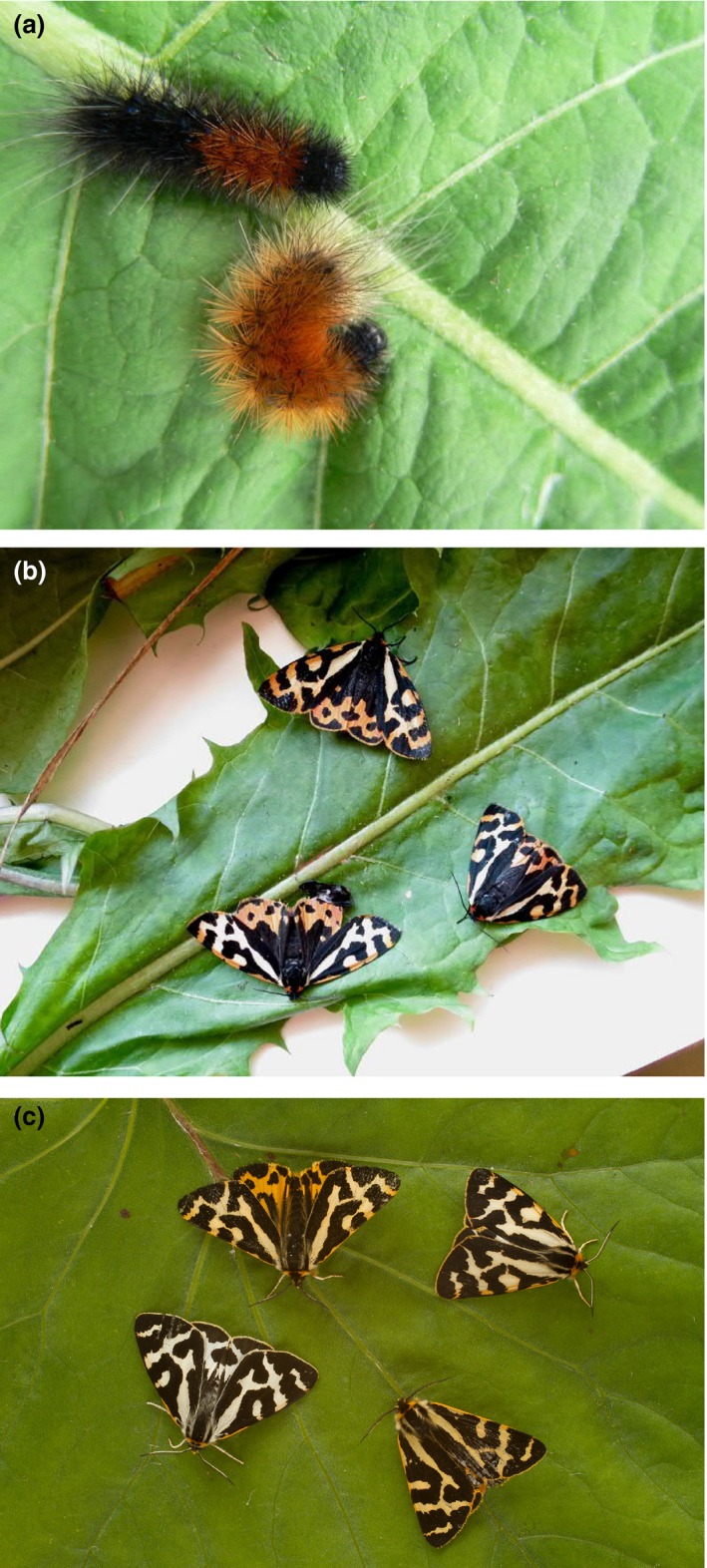
Variation in the (a) larval coloration, (b) female coloration and (c) male coloration in the aposematic wood tiger moth *Arctia plantaginis*

We experimentally tested the life‐history costs of producing and maintaining warning colour pattern under low and high resource (protein content) diets with wood tiger moths *Arctia plantaginis* (Arctiidae) formerly *Parasemia plantaginis* (Rönkä, Mappes, Kaila, & Wahlberg, 2016). The wood tiger moth is an aposematic herbivorous species whose larvae and adults show extensive phenotypic and genetic variation in their warning colour pattern (Lindstedt, Lindström, & Mappes, 2009; Lindstedt et al., 2016; Nokelainen, Lindstedt, & Mappes, 2013; Ojala et al., 2007). Both larval coloration and adult coloration are based on pigments that individuals need to synthesize de novo from protein precursors and energy derived from their diet (Lindstedt, Morehouse, et al., 2010). *Arctia plantaginis* larvae have an orange patch on their otherwise black bodies, and the size of the orange patch varies continuously (Figure 1a). *Arctia plantaginis* moths, the colour patterns of forewings in both females and males, also vary continuously from white to yellow combined with black pattern elements (Figure 1b,c). In Finland, *A. plantaginis* male hindwings are polymorphic and have either yellow or white coloration combined with black patterning (Figure 1c). In females, the coloration of their adult hindwings varies continuously from orange to red with black patterning (Figure 1b).

Based on previous studies, we can assume that in environments of high predation pressure, directional selection by predators should favour larger (Hegna, Nokelainen, Hegna, & Mappes, 2013; Lindstedt et al., 2008) and more salient (Lindstedt et al., 2011; Nokelainen, Hegna, Reudler, Lindstedt, & Mappes, 2012) warning signals in both the larval and adult stages of *A. plantaginis* and thus by default, less melanization surrounding them. However, in colder habitats (Hegna et al., 2013; Lindstedt et al., 2009), under higher risk of bacterial infections (Friman, Lindstedt, Hiltunen, Laakso, & Mappes, 2009; Zhang, Friman, Laakso, & Mappes, 2012), and under higher abundances of naïve predators (Lindstedt et al., 2008; Mappes, Kokko, Ojala, & Lindström, 2014), more melanic (i.e. more cryptic) genotypes should have an advantage. Selective responses for the larger and more salient warning signal patterns can also be genetically constrained as more melanized larvae have more efficient warning signals as an adult females due to negative genetic correlation between larval and female signal efficacy ( Lindstedt et al., 2016) Furthermore, based on earlier studies that have quantified phenotypic and genetic correlations between *A. plantaginis* larval coloration and life‐history traits, we can expect the production of black melanin patterns to be more costly in terms of life‐history traits than large conspicuously coloured orange patterns (Lindstedt et al., 2016; Ojala et al., 2007).

We examined how the life‐history and reproductive costs of producing melanic warning signal types differs on low and high protein diets. We reared larvae from selection lines for small (small proportion of orange with less melanic hairs, large proportion of black with more melanic hairs) and large (large proportion of orange hairs, small proportion of black hairs) warning signal size on artificial diet that contained either low or high amount of protein resources for pigment production. We measured the effect of diet, selection line and their interaction on the amount of melanism in larval and adult coloration, life‐history traits, immune responses and reproductive output. From the adult individuals, we measured the brightness and saturation of wing pigmentation. *Arctia plantaginis* adults do not feed, and therefore, dietary conditions during the larval stage are also critical in terms of adult pigmentation (Lindstedt, Talsma, Ihalainen, Lindström, & Mappes, 2010).

We assumed that if *A. plantaginis* individuals do not prioritize warning colour expression over life‐history traits (or vice versa), low protein content of the diet could decrease the size of black melanin pigmentation (especially for more melanic small signal selection line individuals) as well as performance and immunological responses of individuals. Alternatively, if larvae and adults prioritize their investment in defensive colour pattern early in the resource allocation hierarchy, we may detect only low levels of diet‐induced variation in coloration, but individuals reared on low protein diet may grow slower, have lower immunological responses and produce fewer eggs and offspring (Lindstedt, Talsma, et al., 2010). In both cases, responses to the nutrition conditions could further differ for the larval warning signal genotypes: individuals from the more melanic small signal selection line should pay higher life‐history costs for producing a higher density of melanin pigment in the low protein diet compared with the high protein diet.

## 2 MATERIALS AND METHODS

### 2.1 Study species

*Arctia plantaginis* (Arctiidae) larvae are polyphagous and feed on numerous herbaceous and arborescent plant species (Ojala, Julkunen‐Tiitto, Lindström, & Mappes, 2005). The larvae are hairy and have moderately conspicuous coloration comprising of an orange patch on an otherwise black body. The size of this orange patch is strongly heritable (Lindstedt et al., 2016), but has previously shown to have some level of phenotypic plasticity due to dietary conditions (Ojala et al., 2007). Larvae produce the orange patch by depositing diet‐derived flavonoids and traces of eumelanin in their hairs, and the black colour is based purely on eumelanin (Lindstedt, Morehouse, et al., 2010). The larvae have 5–7 instars, the first two of which are cryptically coloured; orange–black coloration develops at the third instar (Ojala et al., 2007). The pigments deposited in male white and yellow hindwings and orange to red in females are likely to be pheomelanins (J. Kirvesoja, personal communication, 2018).

In Finland, this species usually has one generation per year and *A. plantaginis* typically overwinters as 3rd–4th instar larva. Under laboratory conditions, *A. plantaginis* moths can produce three generations per year and the third‐generation overwinters.

### 2.2 Diet manipulation

To manipulate the protein content of their food, we used an artificial diet that was developed for herbivorous insects (Morehouse & Rutowski, 2010). Low protein resource and high protein resource diet recipes are in Supplementary material (S1). The high protein resource diet contained higher amounts of casein (32.4 g) (predominant source of protein in this diet) than the low protein resource diet (10.8 g). To keep the structure and proportions of other ingredients similar, 21.6 g of cellulose was added to the low protein resource diet. This manipulation affects the energy content of the diet to some extent as the casein component contained 1.5 times more energy in the high than the low protein diet. However, since the casein/cellulose component of the diets formed 16.4% of the diet and all the other dietary components (sugar, cholesterol) are high in energy content, we expect this to have little effect on the overall energy content of low and high protein resource diets. Furthermore, we also allowed larvae to eat artificial food ad libitum throughout the experiment giving them the possibility to compensate for the lower protein and energy content of food by eating more.

### 2.3 Experimental procedure

The experiment entailed a two‐factorial design wherein the factors were larval colour selection lines (small and large orange signals) (described in Reference Lindstedt et al. (2009)) and the resource content of the diet (low and high). For the first 14 days after hatching, larvae were reared in family groups and fed with a mixed diet of lettuce and *Taraxacum* spp. leaves. At the age of 14 days, we randomly selected 10 individuals per family (17 families from the small and 22 families from the large signal line) and divided them into low and high resource diet (5 individuals per diet treatment per family) to control for genetic variation between dietary treatments. For two families from the large signal line, we had 10 individuals per diet treatment per family. In total, we had 85 individuals from the small signal line per low and high protein diet treatment (*N* = 170 in total) and 122 individuals from the large signal line per each diet treatment (*N* = 246 in total). During the experiment, the larvae were reared individually in petri dishes until they reached the adult stage. Individuals were reared in a greenhouse at the University of Jyväskylä in Central Finland (62°N, 26°E) during June and July 2010. The temperature in the greenhouse varied between 20°C and 30°C during the day (approx. 20 hr), and during the night (approx. 4 hr), it decreased to 15–20°C. The larvae were checked daily and fresh food was added ad libitum while removing the old. During the rearing, life‐history traits and colour measurements were recorded for both larvae and adults. In addition, we measured the encapsulation response and antimicrobial activity (zone of inhibition) for all the larvae and reproductive output for adults (see below).

### 2.4 Colour measurements

The proportional size of the orange warning signal patch was measured from the last instar larvae by counting the number of body segments that were covered with orange hairs similarly to Lindstedt et al. (2016). Because larvae always have 13 segments, this measure estimates the proportional size of the orange patch in the larval body. Lower values for orange patch size mean a higher proportion of black melanin coloration, and higher values of orange pattern size mean a lower proportion of black melanin coloration.

Adults were frozen for colour pattern measurements. To measure the size of the melanic patterns of the adults, wings of the dead individuals were gently detached from the body and photographed from both sides using a calibrated Fuji IS digital camera, which records both ultraviolet and human visible signals. The programme was then used to measure and analyse the size of the black patterns (mm^2^) and wing size. Due to mortality during the pupal stage, we had lower number of individuals for the measurements in the adult stage. In total, we measured 10 and 13 females from the small signal line, and 20 and 23 females from the large signal line per low and high protein diet, respectively. For the adult males, we measured 13 and 19 males from the small signal line and 12 and 36 males from the large signal line per low and high protein diets, respectively.

To test how insectivorous birds perceive the possible variation in the brightness and saturation of the colour patterns of wood tiger moths in different treatment groups, hue and brightness of the pattern components were analysed with the Image Calibration and Analysis Toolbox (Troscianko & Stevens, 2015) similar to Lindstedt, Boncoraglio, Cotter, Gilbert, and Kilner (2017). First, the regions of interest (ROIs) from the normalized and linearized images of moths were converted to predicted photoreceptor responses of single and double cone types of a blue tit (Hart, 2001; Hart, Partridge, Cuthill, & Bennett, 2000; Troscianko & Stevens, 2015) by using a mapping function of the Image Calibration and Analysis Toolbox. This mapping is highly accurate compared with reflectance‐based calculations of predicted cone responses (Pike, 2011; Stevens & Cuthill, 2006). Colour vision in birds stems from the four single cone types, while the double cones are likely responsible for luminance‐based tasks (Vorobyev & Osorio, 1998; Vorobyev, Osorio, Bennett, Marshall, & Cuthill, 1998), such as detecting achromatic contrast differences. The vision model converts the ROIs to cone‐catch data, that is to the relative photon catches of a blue tit's four single cones: long wave (LW), medium wave (MW), shortwave (SW) and ultraviolet (UV) cones, as well as to luminance values based on the double cone sensitivity. To analyse the phenotypic and genetic variation in colour of the wood tiger moths, we calculated saturation values (colour richness) similar to Arenas, Walter, and Stevens (2015) and brightness (double cone sensitivity) for the ROIs of the brightly coloured and black patterns in both forewings and hindwings.

### 2.5 Immunological measurements

Encapsulation response (i.e. induced response) is a commonly used method to assess an animal's response against foreign intrusions (e.g. parasitoids) (Schmid‐Hempel, 2005). Encapsulation responses were measured after larvae reached the weight of 100 mg. Larvae were anaesthetized with carbon dioxide (CO_2_), after which two thirds of a sterilized nylon implant (length = 6 mm) was inserted inside the larvae between the second and the third segments from the dorsal side (Friman et al., 2009; Ojala et al., 2005). The immune system of the larvae was allowed to react for 5 hr before the implant was removed by pulling on the implant outside the larva. The resulting encapsulating response was seen as a darkening of the implant. A reaction time of 5 hr was selected because it yields optimal darkening of samples for later analysis with image software. A shorter time would produce too pale and longer times completely blackened samples. The implant was dried and photographed under a microscope with 10× magnification using a Panasonic wv‐CL702 (Panasonic) video recorder. The mean grey value of the implant was measured with imagej software from the 1 mm area from the end being inside the larva. The grey value of the background was subtracted from the grey value of the implant to correct for any variation in lighting during photography. Three measurements were taken from each implant, and their average was used. Higher grey values (darker implant) indicated a stronger response of encapsulation.

Antimicrobial activity against bacterial pathogens was determined using the area of inhibition assay as reported in Nokelainen et al. (2013). As bacteria, we used *Micrococcus luteus* (ATCC strain 4698). We quantified the area of inhibition on the agar plates and used it as a measure of the antimicrobial activity of haemolymph. As a negative control, we added 10 μl of sterile water to the filter paper in the centre of the plate. As a result, we were able to compare whether any produced zones of inhibition were due to antimicrobial activity and not a failure in bacterial growth. The haemolymph sample was taken from the larva at the same time with the implantation by injecting a sterile needle between the second and third segments of the larva, and allowing the haemolymph to form a droplet. Sampling of haemolymph was done before the nylon implant (see above) was inserted, and thereby, antimicrobial activity of haemolymph serves as a measure of constitutive immunity (Schmid‐Hempel, 2005). Similar to the water control, we withdrew 10 μl of haemolymph with a pipette and ejected it onto a filter paper disc on the agar plate. All petri dishes were kept at room temperature (+25°C) for 3 days; after that, the plates were photographed and the area of inhibition was measured using image j software. Area of inhibition was measured by subtracting the maximum area of the inhibition from the area of the filter disc. Area of inhibition was always visible around the filter disc when it occurred, but some individual samples never expressed the area of inhibition. To control the possible error variation among the plates (e.g. differences in bacterial growth), we standardized the antimicrobial activity (i.e. inhibition area of the experimental samples) by subtracting the response of negative water control from it.

### 2.6 Life‐history measurements

Larvae were weighed before they were divided into the diet treatments to minimize differences in weights between the treatment groups. At this point, larvae still had their cryptic coloration; thus, we could not select experimental individuals based on the amount of black in their colour. The individuals were also weighed on the day of their pupation.

After the adults emerged, they were mated within the selection lines and treatments to test whether the number of eggs and number of larvae hatched differed between the treatments or selection lines. We had one male per one female (i.e. one pair per container). Altogether, we had 6 pairs in the small signal line and 8 in the large signal line for the low resource diet and 11 in the small signal line and 20 in the large signal line for the high resource diet.

### 2.7 Statistical analyses

We used generalized linear mixed models (GLMMs) to estimate the treatment effects and family variance within the selection lines on larval traits (signal size, encapsulation response, antimicrobial activity, development time and pupal mass). The selection line and diet, as well as their interaction, were included as fixed factors in the models. To study variation in the sensitivity of families to different diets within the selection lines, and to estimate the presence of family‐by‐diet interactions, we compared models with the following random effect structures: (a) family and (b) family‐by‐diet. The family‐by‐diet interaction was retained in analyses if the fit of the model was improved by their addition, as judged by likelihood ratio tests (model comparison estimates reported in Table S1 and full models with random structures in Table S2, Supplementary material).

For the adult traits (wing size, pattern size, brightness and saturation of colour pattern, number of eggs and number of hatched offspring), we did not have enough individuals per family to reliably estimate family × diet interactions within selection lines. Therefore, all the adult traits were analysed with generalized mixed models where we had selection line and diet as well as their interaction as fixed factors and family as a random factor. We included the size of the forewing and hindwing as a covariate for the analyses on pattern sizes and colour on forewing and hindwings, respectively. Since *A. plantaginis* is sexually dimorphic in terms of adult coloration, we analysed female and male colour traits separately. Pupal size (Lindstedt, Talsma, et al., 2010) was included as a covariate in the analyses of reproductive output traits. The level of significance in all analyses was set at *p* < .05. The Satterthwaite approximation for degrees of freedom was applied when using the function ‘lmer’. All statistical analyses were performed in r studio (v. 1.1.419, 2009–2018 RStudio and packages ‘lmer’ and ‘car’). Dataset of the experiment can be found from Reference Lindstedt, Suisto, and Mappes (2019).

## 3 RESULTS

### 3.1 Effect of diet quality on larval and adult warning coloration

**Figure 2:**
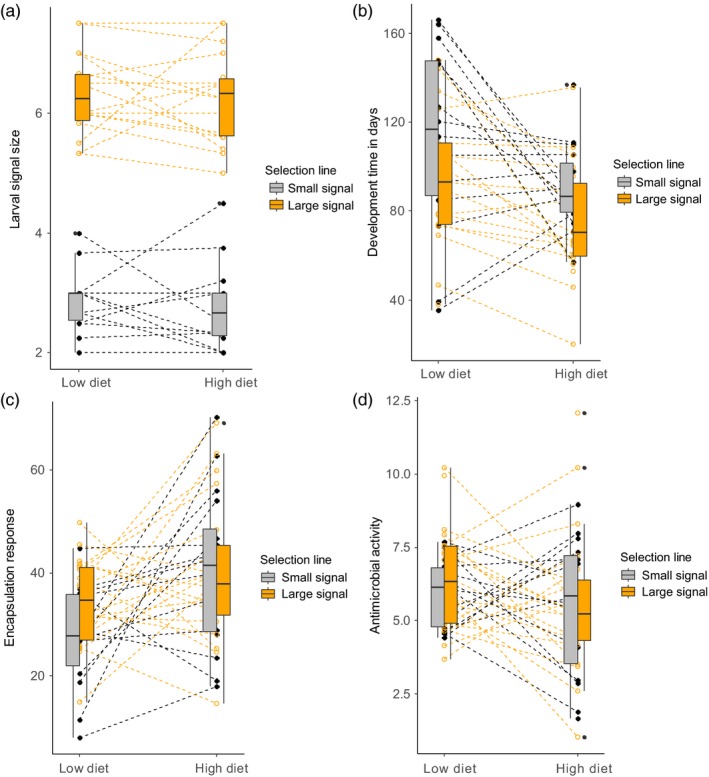
Dashed lines show the family means for the (a) larval signal size, (b) development time, (c) encapsulation rate and (d) antimicrobial activity based on the raw data (i.e. each dashed line represents a family). Box plots show the mean values for the small (grey) and large (orange) signal lines on low protein and high protein diet treatments

Larvae were more melanic (i.e. orange warning signals of larvae were smaller) in the small signal line than in the large signal line (*F*_1;35_ = 287.88, *p* < .001). However, the protein content of the diet did not have any significant effects on larval coloration (*F*_1;176.5_ = 0.00, *p* = .985). There were no significant interactions between the signal line and diet (*F*_1,176.5_ = 0.030, *p* = .864; Figure 2a). Family‐level variation within selection lines explained 23% of all the phenotypic variation in the larval signal line (Table S2).

**Figure 3:**
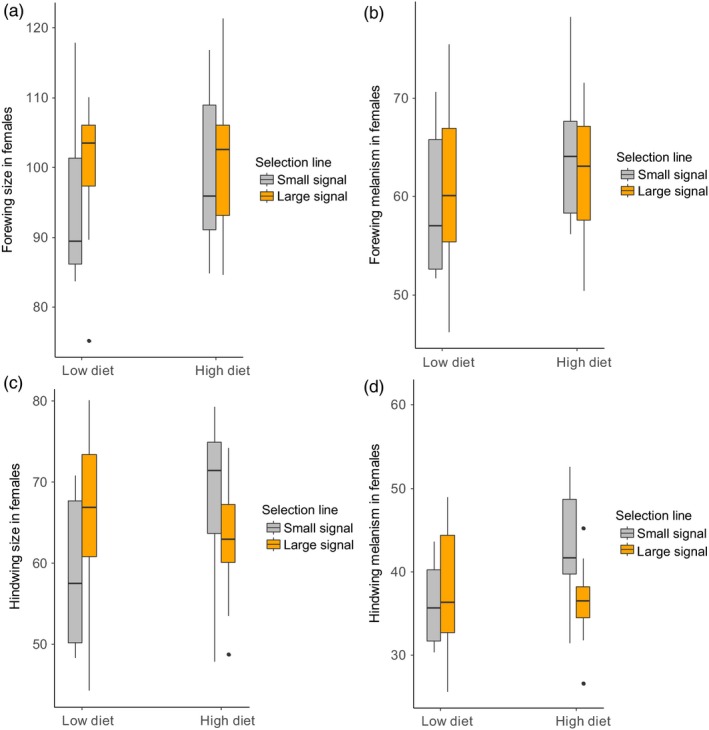
Box plots show the mean values for the (a) forewing size, (b) forewing melanism, (c) hindwing size and (d) hindwing melanism for the small (grey) and large (orange) signal lines on low protein and high protein diet treatments in *Arctia plantaginis* female moths

**Figure 4:**
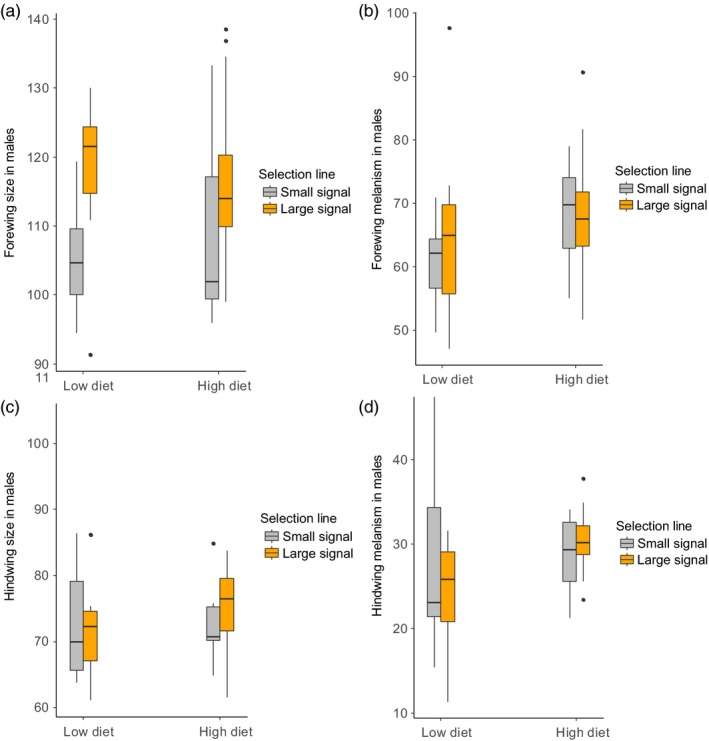
Box plots show the mean values for the (a) forewing size, (b) forewing melanism, (c) hindwing size and (d) hindwing melanism for the small (grey) and large (orange) signal lines on low protein and high protein diet treatments in *Arctia plantaginis* male moths

The size of the melanic patterns in adult's forewings was not affected by diet, signal line or their interactions in females (diet: *F*_1;48.6_ = 0.169, *p* = .683, signal line: *F*_1;24.5_ = 1.292, *p* = .267, diet * signal line: *F*_1;48.6_ = 0.265, *p* = .609; Figure 3b). Female moths with larger forewings had larger melanic patterns (*F*_1;48.6_ = 0.265, *p* < .001). In males, forewings had larger melanic patterns on high protein diet than on low protein diet (*F*_1;48.8_ = 4.050, *p* = .050; Figure 4b). Signal line (*F*_1;28.7_ = 1.443, *p* = .239) and signal line and diet interactions (*F*_1;48.7_ = 0.106, *p* = .747) did not significantly affect forewing melanism. Similar to females, males with larger forewings had larger melanic patterns (*F*_1;48.7_ = 65.051, *p* < .001).

The protein content of the diet did not significantly affect hindwing melanism in females (*F*_1;47.8_ = 0.308, *p* = .582; Figure 3d). However, females from the more melanic small larval signal line had more melanic hindwings than females from the large signal line (*F*_1;20.6_ = 0.308, *p* = .027). There were no significant interactions between the diet and signal line (*F*_1;47.0_ = 0.608, *p* = .440). Females with larger hindwings had larger melanic patterns (*F*_1;36.6_ = 98.351, *p* < .001). For males, diet (*F*_1;46.4_ = 1.981, *p* = .166) and selection line (*F*_1;24.1_ = 0.061, *p* = .808) did not significantly affect hindwing melanism in adults (Figure 4d). Nor were there any interactions (*F*_1;46.9_ = 2.262, *p* = .139). In general, patterns were generally larger in larger sized hindwings (*F*_1;50.0_ = 14.648, *p* < .001).

Diet, selection line and their interactions did not have significant effects on the brightness (double cone value) of forewing colour patterns in females and males (All *p*‐values > .245, Table S3, Supplementary material). However, colour patterns in the forewings of females were brighter when their size was smaller (*p* = .037, Table S3).

The brightness of hindwings in females and males was not affected by diet, signal line or their interaction (all *p*‐values > .260, Table S3). Again, males (*p* < .001) and females (*p* = .022) with smaller hindwings had brighter coloration (Table S3). The brightness of black melanic patterns in forewings and hindwings was not affected by the wing size and did not differ among diets, selection lines or due to their interactions in either of the sexes (all *p*‐values > .057, Table S3).

Diet, signal line, or their interactions did not have a significant effect on the saturation of forewing colour patterns (all *p*‐values > .145, Table S4, Supplementary material). Neither was there a significant effect of forewing size on the saturation of light pattern elements (all *p*‐values > .063, Table S4). The saturation of colour patterns of hind wing colour did not differ between the diet, signal line and their interactions (all *p*‐values > .381, Table S4). However, males with smaller hindwings had more saturated (yellower) colour (*p* = .002, Table S4).

Females from small signal line had less saturated black pigmentation on their forewings than females from large signal line (*p* = .027, Table S4). However, diet or its interaction with signal line did not significantly affect the saturation of the black patterns in females (all *p*‐values > .504, Table S4). Diet, signal line, their interactions or hindwing size did not significantly affect the saturation of black pigmentation in the female hindwings (all *p*‐values > .138, Table S4). For the males, diet, signal line or their interactions did not significantly affect the saturation of black pattern elements in forewings or hindwings (all *p*‐values > .151, Table S4), nor did the wing size (Table S4).

### 3.2 Effect of diet quality on immunology

Diet had a significant effect on immunological defence: encapsulation response was higher on the high protein diet than on the low protein diet (*F*_1,222_ = 8.090, *p* = .005; Figure 2c). Encapsulation response did not differ between small and large signal selection lines (*F*_1,222_ = 1.056, *p* = .305). Nor were there any significant interactions between the diet and signal line (*F*_1,222_ = 2.061, *p* = .153). 0% of the variation in encapsulation response was explained by family‐level variation within selection lines (Table S2).

Antimicrobial activity did not differ significantly between the low and high protein diets (*F*_1;185.4_ = 1.746, *p* = .188) or small and large signal lines (*F*_1;29.9_ = 0.054, *p* = .818; Figure 2d). There were no significant interactions between the signal line and diet (*F*_1;185.4_ = 1.459, *p* = .229).

### 3.3 Effect of diet quality on life‐history traits and reproductive output

Pupal mass did not differ significantly on low and high protein diets (*F*_1;168.5_ = 1.575, *p* = .211). Pupae from the small signal line were heavier than pupae from the large signal line (*F*_1;27.1_ = 4.318, *p* = .047). There was no significant interaction between the diet and selection line (*F*_1;168.5_ = 0.280, *p* = .597).

In the adult stage, there was a significant interaction between the protein content of the diet and larval signal line on hindwing size in females (*F*_1,49_ = 5.352, *p* = .025): on the low protein diet, individuals originating from the more melanic larval signal line had smaller hindwings than individuals originating from the large signal line (Figure 2c). On the high protein diet, the difference was smaller and the trend reversed as individuals from the small signal line tended to have larger hindwings than individuals from the large signal line. Among males, signal line (*F*_1;25.0_ = 0.183, *p* = .672) or diet (*F*_1;47.0_ = 0.040, *p* = .842) and their interaction (*F*_1;47.0_ = 0.752, *p* = .390) did not affect hindwing size (Figure 3c).

Female forewing size did not differ significantly between diet treatments (*F*_1,50_ = 0.166, *p* = .686), signal lines (*F*_1,50_ = 2.144, *p* = .489) or due to their interaction (*F*_1,50_ = 0.486, *p* = .489; Figure 3a). In males, diet (*F*_1;50.0_ = 0.002, *p* = .966) or its interaction with signal line on forewing size did not differ significantly (*F*_1;50.0_ = 0.953, *p* = .334). However, males originating from the large signal line had larger forewings than small signal line males (*F*_1,25.9_ = 5.608, *p* = .026; Figure 4a).

There were no significant interactions between diet and selection line in development time from larva to pupa (*F*_1;22.4_ = 0.022, *p* = .885), and it was omitted from the final model. Development time was shorter on the high protein diet than on the low protein diet (*F*_1;22.8_ = 6.874, *p* = .015; Figure 2b). Individuals also developed faster in the large signal selection line compared with the small signal selection line (*F*_1;32.2_ = 4.680, *p* = .038). The proportion of random phenotypic variation in development time explained by family was 40% and by diet 16% (Table S2).

Selection line, diet or their interaction did not have any significant effects on the number of eggs (signal line: *F*_1;26.7_ = 1.559, *p* = .223; diet: *F*_1;73.1_ = 0.042, *p* = .838; signal line * diet: *F*_1;72.6_ = 0.127, *p* = .723) and number of offspring produced (signal line: *F*_1;31.6_ = 0.910, *p* = .347; diet: *F*_1;78.1_ = 0.318, *p* = .575; signal line * diet: *F*_1;77.8_ = 0.521, *p* = .473). Pupal mass did not explain the reproductive output (number of eggs: *F*_1;68.7_ = 2.880, *p* = .094; offspring number: *F*_1;72.7_ = 3.968, *p* = .473).

## 4 DISCUSSION

How individuals allocate limited resources among different traits is often genetically determined. Traits that play critical role in terms of individual fitness might be genetically prioritized in investment instead of decreasing the general performance in all of the traits when nutritional conditions are poor. Our findings suggest that nutritional conditions do not affect warning signal expression (orange signal size in the larval stage and bright hindwing coloration in the adult stage) in generalist, herbivorous aposematic *A. plantaginis* moths, but instead low protein content of the diet decreases their immune defence, development time and female's wing size. Furthermore, individuals originating from the more melanic (small warning signal) larval signal line paid higher costs especially under low protein diet in terms of development time and female hindwing size. Development time was also the only measured trait in which families varied in their responses for the dietary conditions (diet‐by‐family interaction). In terms of other life‐history and immunological traits, families showed little variation in family‐level plasticity. Altogether, these results suggest that in both the larval and adult stages individuals may prioritize investment in their defensive colour pattern in comparison with performance and size‐associated traits under poor nutritional conditions.

Our results are somewhat conflicting with previous studies that have shown nutrition to affect melanization in cryptically coloured insects (Lee et al., 2008). We found some direct effects of dietary conditions on the melanin pigmentation in males, whose forewings were more melanized on the high protein diet than on the low protein diet. When *A. plantaginis* moths are resting, hindwings are hidden under the forewings. The function of forewings has not been tested yet, but they are likely to play an important role not only in protective coloration but also in thermoregulation. However, diet had little effect on the signal size in larval stage as well as colour and patterning in adult hindwings which play important role in warning signalling in this species (Lindstedt et al., 2011; Nokelainen et al., 2012). Neither was there variation in plasticity of signal traits between signal lines or among families (diet‐by‐family interaction). However, this is not perhaps so surprising as warning signal expression can be expected to be quite robust to variation in growth condition as it ensures effective protection against predators. Our results here, together with those of earlier studies, are in accordance with this expectation: the size and colour of the bright signal pattern are strongly inherited in *A. plantaginis* (Lindstedt et al., 2016; Nokelainen et al., 2013) and show very little environmental plasticity (Lindstedt et al., 2009; Lindstedt, Talsma, et al., 2010; Nokelainen et al., 2013). Thus, the role of diet‐induced plasticity in the maintenance of colour variation is likely to play a minor role compared with variation in strength and direction of selection pressures on different signal genotypes (Hegna et al., 2013; Lindstedt et al., 2008; Nokelainen et al., 2012). Interestingly, our results suggest that individuals may even be able to compensate for the smaller hindwing size under low protein diet by consequently increasing the density of pigmentation: in adults, the brightness and saturation of hind wing warning signals increased when the size of the hindwing decreased.

We did not find a clear trade‐off between investment in immune defence and the production of melanin pigmentation that is found in other studies (Lee et al., 2008). The encapsulation response in insects is a cell‐mediated immunological response that is linked to the phenoloxidase (PO) cascade similar to melanization (e.g. Cotter, Hails, Cory, & Wilson, 2004; Wilson, Cotter, Reeson, & Pell, 2001). As both of these processes need amino acids for precursors, the low protein content in the diet may limit their efficacy. In this study, we only found a significant effect of dietary protein content on the encapsulation response, but individuals from the more melanic signal selection line did not have significantly lower responses on the low resource diet. The cost of immune defence in low protein diet is likely to be a result of competition between precursors associated with different physiological processes (Blount, Speed, Ruxton, & Stephens, 2009; Stoehr, 2006).

Neither did we find any effect of nutrition or signal selection line on antimicrobial activity. Unlike melanin pigment synthesis and encapsulation, antimicrobial activity is based on the humoural immune response and should not be directly linked to the phenoloxidase cascade (Cotter, Kruuk, & Wilson, 2004; Cotter, Myatt, Benskin, & Wilson, 2008). Thus, it is possible that on the low protein resource diet individuals have more resources to allocate to antimicrobial activity than to the encapsulation response. Since larvae were allowed to feed ad libitum during the experiment, it is also possible that larvae from the more melanic small signal line were able to increase protein intake to some extent on low protein diet. Thus, they may have been able to compensate protein costs of encapsulation response and antimicrobial defence to some extent (Povey, Cotter, Simpson, Lee, & Wilson, 2009). Future studies considering the trade‐off between immune responses and melanization should measure phenoloxidase (PO) activity as it is more directly linked to melanin synthesis than measurements used here.

Our previous research has shown that production of melanic coloration in the larval stage is more costly as higher amounts of black coloration in the larval stage are negatively genetically correlated with development time in *A. plantaginis* (Lindstedt et al., 2016). Our results here are in accordance with this finding as development time was longest for individuals who originated from the small warning signal line and were reared under low protein diet (Figure 2b). Similarly, females who originated from the small signal line had smaller hindwings under low protein diet than females from the large signal line. Longer development time during the larval stage has suggested to be costly as it can increase vulnerability to predation and parasitism (Damman, 1987; Teder & Tammaru, 2001). Since we did not find differences in pupal sizes due to diet, and individuals from the small signal line were actually a little heavier than individuals from the large signal line, our results suggest that individuals were able to compensate for production costs of melanin pigmentation on the low protein diet by developing longer but to similar sizes. Fitness consequences of small hindwings in females are more difficult to interpret, but it may affect their ability to disperse or escape from predators. Since pupal mass and reproductive output did not differ significantly among diets, it is possible that females from the small signal line allocated more to egg production than to size under the low protein diet.

More generally, our results suggest that availability of proteins in the diet does not constrain signal efficacy (i.e. larger and more salient pattern sizes), but it can slow down selective responses on increased melanism (Lindstedt et al., 2016; Ojala et al., 2005, 2007). This can happen directly by constraining the melanism in forewings or indirectly via higher life‐history costs of producing melanic pattern under a low protein diet. These constraints on melanin can have important fitness consequences: *A. plantaginis* occurs only in the northern hemisphere, and it has populations in very high latitudes and mountain regions (Hegna, Galarza, & Mappes, 2015; Hegna et al., 2013). As they are ectotherms, solar radiation is their primary source of heat gain. In addition, *A. plantaginis* also overwinters in the larval stage. High melanism in larvae has been shown to be adaptive in colder habitats as it increases growth efficiency (Lindstedt et al., 2009) and decreases conspicuousness and attack risk for naïve predators (Lindstedt et al., 2008; Mappes et al., 2014). Also in adults, individuals are darker in high latitudes and warm up more quickly (Hegna et al., 2013). Therefore, they can escape faster from predators, and fly longer distances, giving them more time to find food, suitable mates and suitable oviposition sites on which to lay their eggs.

In conclusion, our results indicate that variation in the amount of dietary precursors needed for melanin synthesis can have multiple fitness consequences for the insects; it affects their immunological responses, wing size in females, and prolongs their development time to the reproductive adult stage. Perhaps most interestingly, our results suggest that the development of warning signal patterns under varying environmental conditions is likely to be well conserved either developmentally or in terms of resource allocation between signal and life‐history traits. An interesting avenue for future research will be to examine how aposematic organisms prioritize the investment in warning signal pattern and toxicity under nutritional stress (Burdfield‐Steel et al., 2018; Holloway, de Jong, & Ottenheim, 1993; de Jong, Holloway, Brakefield, & de Vos, 1991) and how these trade‐offs shape the information content of warning signals. This is especially intriguing question to study when organisms produce defensive compounds by themselves, making it energetically costly and potentially dependent on the nutrition content of the diet.

## AUTHORS' CONTRIBUTIONS

C.L. and J.M. conceived the ideas and designed methodology; C.L. and K.S. collected the data; C.L. analysed the data; and C.L. led the writing of the manuscript. All authors contributed critically to the drafts and gave final approval for publication.

## Data Availability

All the datasets used in these analyses can be found in the Dryad Digital Repository: https://doi.org/10.5061/dryad.bj7bg83 (Lindstedt et al., 2019).
