# Supplementary material for: Appearance before performance? Nutritional constraints on life‐history traits, but not warning signal expression in aposematic moths
Source: J Anim Ecol. 2019 Oct 23;89(2):494–505. doi: 10.1111/1365-2656.13103 (PMC7027542; doi:10.1111/1365-2656.13103)
Supplement: Supplementary file 1 [file JANE-89-494-s001.doc]

**Supplementary material.**

**Appearance before performance? Nutritional constraints on life-history traits, but not warning signal expression in aposematic moths**

Lindstedt, Carita^1^; Suisto, Kaisa^1^ and Mappes, Johanna^1^

1) Centre of Excellence in Biological Interactions, Department of Biological and Environmental Sciences, University of Jyväskylä, Finland

Diet treatments with low and high protein availability

Notice that the casein used in this diet is Teklad Vitamin-Free. This is important, as casein is the predominant source of protein in this diet, and is used to vary the protein availability. If regular casein is used, the availability of a number of vitamins will also vary, introducing a potential dietary confound.

| ***Hi N*** |  |  | ***Lo N*** |  |  |
| --- | --- | --- | --- | --- | --- |
| Agar | g | 18 | Agar | g | 18 |
| Wheat Germ | g | 72 | Wheat Germ | g | 72 |
| Teklad VitFree Casein | g | ***32.4*** | Teklad VitFree Casein | g | ***10.8*** |
| Sucrose | g | 28.8 | Sucrose | g | 28.8 |
| Salt Mix | g | 10.8 | Salt Mix | g | 10.8 |
| Torula Yeast | g | 14.4 | Torula Yeast | g | 14.4 |
| Cellulose | g | 0 | Cellulose | g | ***21.6*** |
| Cholesterol | g | 3.15 | Cholesterol | g | 3.15 |
| Sorbic Acid | g | 1.8 | Sorbic Acid | g | 1.8 |
| Methylparaben | g | 1.2 | Methylparaben | g | 1.2 |
| Ascorbic Acid | g | 3.6 | Ascorbic Acid | g | 3.6 |
| VanderZandt Vit Mix | g | 12.6 | VanderZandt Vit Mix | g | 12.6 |
| Aureomycin | g | 1.2 | Aureomycin | g | 1.2 |
| Water (cool) | ml | 450 | Water (cool) | ml | 450 |
| Water (boiling) | ml | 450 | Water (boiling) | ml | 450 |
| Linseed oil | ml | 6 | Linseed oil | ml | 6 |

Table S1. Likelihood ratio test comparisons of GLMMs with different random effect structures regarding signal and life-history traits of *A. plantaginis* larvae. The model used in the statistical analyses is illustrated by *, and the others are sorted by their log likelihood values. P-values refer to the likelihood ratio test comparisons of each model to the one above them. Table shows the GLMMs used in the statistical analyses and therefore illustrates the presence or absence of family-by-treatment interactions (suggesting G x E interactions) in all studied traits.

| **Response variable and models with different random effects** | **log likelihood** | **df** | **p** |
| --- | --- | --- | --- |
|  |  |  |  |
| *Larval signal size* |  |  |  |
| Family* | -255.88 | 6 |  |
| Family + Diet | -254.27 | 8 | 0.199 |
|  |  |  |  |
| *Development time* |  |  |  |
| Family | -869.17 | 6 |  |
| Family + Diet* | -865.99 | 8 | 0.042 |
|  |  |  |  |
| *Encapsulation* |  |  |  |
| Family* | -989.81 | 6 |  |
| Family + Diet | -988.48 | 8 | 0.263 |
|  |  |  |  |
| *Antimicrobial activity* |  |  |  |
| Family* |  |  |  |
| Family + Diet did not converge |  |  |  |
|  |  |  |  |
| *Pupa mass* |  |  |  |
| Family* | -964.43 | 6 |  |
| Family + Diet | -963.92 | 8 | 0.599 |

Table S2. Full summary statistics for the influence of fixed (diet quality, signal line and their interaction), random (family (all traits) and its interaction with diet (family-by-diet) quality for larval traits) effects on the studied traits of *A. plantaginis* individuals larvae and adults. Significant effects of fixed factors are illustrated with * (p < 0.05). See Table S1 for the GLMMs used in the analyses.

| *Larval traits* |  |  |  |  |
| --- | --- | --- | --- | --- |
|  |  |  |  |  |
| **Signal size** |  |  |  |  |
| (n = 190, families = 35) |  |  |  |  |
|  |  |  |  |  |
| Random effects |  |  |  |  |
| **Source of variation** | **Name** | **σ2** | **SD** |  |
| Family | Intercept | 0.210 | 0.458 |  |
| Residual |  | 0.718 | 0.847 |  |
|  |  |  |  |  |
| Fixed effects |  |  |  |  |
| **Source of variation** | **Estimate** | **s.e.** | **t** | **P** |
| Intercept | 2.746 | 0.196 | 14.013 | < 0.001 |
| Diet quality | 0.026 | 0.208 | 0.124 | 0.902 |
| Signal line | 3.495 | 0.256 | 13.652 | <0.001 |
| Diet * Signal line | -0.046 | 0.270 | -0.172 | 0.864 |
|  |  |  |  |  |
| **Development time** |  |  |  |  |
| (n = 177, families = 35) |  |  |  |  |
|  |  |  |  |  |
| Random effects |  |  |  |  |
| **Source of variation** | **Name** | **σ2** | **SD** | **Corr** |
| Family | Intercept | 876.700 | 29.610 |  |
|  | Diet | 345.800 | 18.600 | -0.940 |
| Residual |  | 947.200 | 30.780 |  |
|  |  |  |  |  |
| Fixed effects |  |  |  |  |
| **Source of variation** | **Estimate** | **s.e.** | **t** | **P** |
| Intercept | 110.720 | 9.879 | 11.208 | < 0.001 |
| Diet quality | -16.964 | 9.357 | -1.813 | 0.083 |
| Signal line | -18.168 | 13.229 | -1.373 | 0.181 |
| Diet * Signal line | 1.829 | 12.468 | 0.147 | 0.885 |
|  |  |  |  |  |
| **Encapsulation** |  |  |  |  |
| (n = 226, families = 37) |  |  |  |  |
|  |  |  |  |  |
| Random effects |  |  |  |  |
| **Source of variation** | **Name** | **σ2** | **SD** |  |
| Family | Intercept | 0.000 | 0.000 |  |
| Residual |  | 406.300 | 20.160 |  |
|  |  |  |  |  |
| Fixed effects |  |  |  |  |
| **Source of variation** | **Estimate** | **s.e.** | **t** | **P** |
| Intercept | 28.379 | 3.110 | 9.124 | < 0.001 |
| Diet quality | 11.759 | 4.280 | 2.747 | 0.007 |
| Signal line | 6.768 | 3.979 | 1.701 | 0.090 |
| Diet * Signal line | -7.888 | 5.495 | -1.436 | 0.153 |
|  |  |  |  |  |
| **Antimicrobial activity** |  |  |  |  |
| (n = 200, families = 36) |  |  |  |  |
|  |  |  |  |  |
| Random effects |  |  |  |  |
| **Source of variation** | **Name** | **σ2** | **SD** |  |
| Family | Intercept | 0.447 | 0.668 |  |
| Residual |  | 7.979 | 2.825 |  |
|  |  |  |  |  |
| Fixed effects |  |  |  |  |
| **Source of variation** | **Estimate** | **s.e.** | **t** | **P** |
| Intercept | 5.934 | 0.485 | 12.229 | < 0.001 |
| Diet quality | -0.047 | 0.638 | -0.073 | 0.942 |
| Signal line | 0.608 | 0.625 | 0.972 | 0.334 |
| Diet * Signal line | -0.997 | 0.825 | -1.208 | 0.229 |
|  |  |  |  |  |
| **Pupal mass** |  |  |  |  |
| (n = 180, families = 35) |  |  |  |  |
|  |  |  |  |  |
| Random effects |  |  |  |  |
| **Source of variation** | **Name** | **σ2** | **SD** |  |
| Family | Intercept | 323.400 | 17.980 |  |
| Residual |  | 2843.900 | 53.330 |  |
|  |  |  |  |  |
| Fixed effects |  |  |  |  |
| **Source of variation** | **Estimate** | **s.e.** | **t** | **P** |
| Intercept | 276.097 | 11.356 | 24.313 | < 0.001 |
| Diet quality | -6.269 | 13.473 | -0.465 | 0.642 |
| Signal line | -17.430 | 14.710 | -1.185 | 0.240 |
| Diet * Signal line | -9.147 | 17.282 | -0.529 | 0.597 |
|  |  |  |  |  |
|  |  |  |  |  |
| *Adult traits* |  |  |  |  |
|  |  |  |  |  |
| **Forewing melanism in females** |  |  |  |  |
| (n = 54, families = 31) |  |  |  |  |
|  |  |  |  |  |
| Random effects |  |  |  |  |
| **Source of variation** | **Name** | **σ2** | **SD** |  |
| Family | Intercept | 24.160 | 4.915 |  |
| Residual |  | 18.160 | 4.262 |  |
|  |  |  |  |  |
| Fixed effects |  |  |  |  |
| **Source of variation** | **Estimate** | **s.e.** | **t** | **P** |
| Intercept | 2.869 | 6.077 | 0.472 | 0.639 |
| Diet quality | 1.584 | 2.977 | 0.532 | 0.597 |
| Signal line | -1.661 | 2.867 | -0.579 | 0.566 |
| Forewing size | 0.594 | 0.059 | 10.129 | <0.001 |
| Diet * Signal line | -1.767 | 3.433 | -0.515 | 0.609 |
|  |  |  |  |  |
| **Forewing melanism in males** |  |  |  |  |
| (n = 54, families = 27) |  |  |  |  |
|  |  |  |  |  |
| Random effects |  |  |  |  |
| **Source of variation** | **Name** | **σ2** | **SD** |  |
| Family | Intercept | 10.170 | 3.189 |  |
| Residual |  | 46.850 | 6.845 |  |
|  |  |  |  |  |
| Fixed effects |  |  |  |  |
| **Source of variation** | **Estimate** | **s.e.** | **t** | **P** |
| Intercept | -4.871 | 8.312 | -0.586 | 0.561 |
| Diet quality | 5.140 | 3.251 | 1.581 | 0.120 |
| Signal line | -2.364 | 3.772 | -0.627 | 0.534 |
| Forewing size | 0.620 | 0.077 | 8.065 | <0.001 |
| Diet * Signal line | -1.440 | 4.432 | -0.325 | 0.747 |
|  |  |  |  |  |
| **Hindwing melanism in females** |  |  |  |  |
| (n = 50, families = 30) |  |  |  |  |
|  |  |  |  |  |
| Random effects |  |  |  |  |
| **Source of variation** | **Name** | **σ2** | **SD** |  |
| Family | Intercept | 7.445 | 2.729 |  |
| Residual |  | 19.316 | 4.395 |  |
|  |  |  |  |  |
| Fixed effects |  |  |  |  |
| **Source of variation** | **Estimate** | **s.e.** | **t** | **P** |
| Intercept | 1.254 | 4.016 | 0.312 | 0.756 |
| Diet quality | 2.078 | 2.631 | 0.790 | 0.434 |
| Signal line | -2.823 | 2.425 | -1.164 | 0.253 |
| Hindwing size | 0.600 | 0.060 | 9.917 | <0.001 |
| Diet * Signal line | -2.481 | 3.183 | -0.780 | 0.440 |
|  |  |  |  |  |
| **Hindwing melanism in males** |  |  |  |  |
| (n = 53, families = 27) |  |  |  |  |
|  |  |  |  |  |
| Random effects |  |  |  |  |
| **Source of variation** | **Name** | **σ2** | **SD** |  |
| Family | Intercept | 9.284 | 3.047 |  |
| Residual |  | 24.569 | 4.957 |  |
|  |  |  |  |  |
| Fixed effects |  |  |  |  |
| **Source of variation** | **Estimate** | **s.e.** | **t** | **P** |
| Intercept | 6.202 | 5.973 | 1.038 | 0.304 |
| Diet quality | -0.178 | 2.497 | -0.071 | 0.943 |
| Signal line | -3.005 | 2.824 | -1.064 | 0.293 |
| Hindwing size | 0.294 | 0.077 | 3.827 | <0.001 |
| Diet * Signal line | 5.041 | 3.352 | 1.504 | 0.139 |
|  |  |  |  |  |
| **Brightness of colourful patterns in female's forewings** | |  |  |  |
| (n = 21, families = 15) |  |  |  |  |
|  |  |  |  |  |
| Random effects |  |  |  |  |
| **Source of variation** | **Name** | **σ2** | **SD** |  |
| Family | Intercept | 0.000 | 0.000 |  |
| Residual |  | 4407967.000 | 2100.000 |  |
|  |  |  |  |  |
| Fixed effects |  |  |  |  |
| **Source of variation** | **Estimate** | **s.e.** | **t** | **P** |
| Intercept | 39520.730 | 5756.730 | 6.865 | <0.001 |
| Diet quality | -1650.500 | 1937.290 | -0.852 | 0.410 |
| Signal line | -211.350 | 1692.270 | -0.125 | 0.903 |
| Forewing size | -122.950 | 52.670 | -2.334 | 0.037 |
| Diet * Signal line | 585.900 | 2195.360 | 0.267 | 0.794 |
|  |  |  |  |  |
| **Brightness of colourful patterns in male's forewings** | |  |  |  |
| (n = 24, families = 19) |  |  |  |  |
|  |  |  |  |  |
| Random effects |  |  |  |  |
| **Source of variation** | **Name** | **σ2** | **SD** |  |
| Family | Intercept | 7885667.000 | 2808.000 |  |
| Residual |  | 3988709.000 | 1997.000 |  |
|  |  |  |  |  |
| Fixed effects |  |  |  |  |
| **Source of variation** | **Estimate** | **s.e.** | **t** | **P** |
| Intercept | 15347.260 | 6193.450 | 2.478 | 0.020 |
| Diet quality | -409.170 | 2320.620 | -0.176 | 0.863 |
| Signal line | 2432.500 | 2223.030 | 1.094 | 0.287 |
| Forewing size | 106.680 | 55.660 | 1.917 | 0.065 |
| Diet * Signal line | -93.300 | 2610.660 | -0.036 | 0.972 |
|  |  |  |  |  |
| **Brightness of colourful patterns in female's hindwings** | |  |  |  |
| (n = 21, families = 15) |  |  |  |  |
|  |  |  |  |  |
| Random effects |  |  |  |  |
| **Source of variation** | **Name** | **σ2** | **SD** |  |
| Family | Intercept | 4755982.000 | 2181.000 |  |
| Residual |  | 1368053.000 | 1170.000 |  |
|  |  |  |  |  |
| Fixed effects |  |  |  |  |
| **Source of variation** | **Estimate** | **s.e.** | **t** | **P** |
| Intercept | 19975.348 | 3026.420 | 6.600 | <0.001 |
| Diet quality | 1250.861 | 2270.111 | 0.551 | 0.591 |
| Signal line | 2965.199 | 1939.224 | 1.529 | 0.150 |
| Hindwing size | -113.954 | 40.563 | -2.809 | 0.033 |
| Diet * Signal line | -2817.821 | 2410.001 | -1.169 | 0.261 |
|  |  |  |  |  |
| **Brightness of colourful patterns in male's hindwings** | |  |  |  |
| (n = 24, families = 19) |  |  |  |  |
|  |  |  |  |  |
| Random effects |  |  |  |  |
| **Source of variation** | **Name** | **σ2** | **SD** |  |
| Family | Intercept | 0.000 | 0.000 |  |
| Residual |  | 24319713.000 | 4932.000 |  |
|  |  |  |  |  |
| Fixed effects |  |  |  |  |
| **Source of variation** | **Estimate** | **s.e.** | **t** | **P** |
| Intercept | -11244.440 | 11146.420 | -1.009 | 0.317 |
| Diet quality | -2049.070 | 3356.680 | -0.610 | 0.544 |
| Signal line | -3779.950 | 3378.210 | -1.119 | 0.267 |
| Hindwing size | 558.320 | 160.680 | 3.475 | <0.001 |
| Diet * Signal line | 4034.270 | 4307.080 | 0.937 | 0.352 |
|  |  |  |  |  |
| **Brightness of black in female's forewings** |  |  |  |  |
| (n = 21, families = 15) |  |  |  |  |
|  |  |  |  |  |
| Random effects |  |  |  |  |
| **Source of variation** | **Name** | **σ2** | **SD** |  |
| Family | Intercept | 292457.000 | 540.800 |  |
| Residual |  | 79118.000 | 281.300 |  |
|  |  |  |  |  |
| Fixed effects |  |  |  |  |
| **Source of variation** | **Estimate** | **s.e.** | **t** | **P** |
| Intercept | 5833.255 | 1119.145 | 5.212 | 0.005 |
| Diet quality | 292.839 | 558.925 | 0.524 | 0.613 |
| Signal line | 103.244 | 484.436 | 0.213 | 0.835 |
| Forewing size | -28.937 | 9.780 | -2.959 | 0.057 |
| Diet * Signal line | 8.956 | 592.458 | 0.015 | 0.988 |
|  |  |  |  |  |
| **Brightness of black in male's forewings** |  |  |  |  |
| (n = 24, families = 19) |  |  |  |  |
|  |  |  |  |  |
| Random effects |  |  |  |  |
| **Source of variation** | **Name** | **σ2** | **SD** |  |
| Family | Intercept | 322327.000 | 567.700 |  |
| Residual |  | 261124.000 | 511.000 |  |
|  |  |  |  |  |
| Fixed effects |  |  |  |  |
| **Source of variation** | **Estimate** | **s.e.** | **t** | **P** |
| Intercept | 3998.953 | 1420.799 | 2.815 | 0.011 |
| Diet quality | -390.034 | 514.552 | -0.758 | 0.460 |
| Signal line | 202.100 | 502.896 | 0.402 | 0.692 |
| Forewing size | -5.832 | 12.804 | -0.455 | 0.654 |
| Diet * Signal line | 231.570 | 597.029 | 0.388 | 0.703 |
|  |  |  |  |  |
| **Brightness of black in female's hindwings** |  |  |  |  |
| (n = 21, families = 15) |  |  |  |  |
|  |  |  |  |  |
| Random effects |  |  |  |  |
| **Source of variation** | **Name** | **σ2** | **SD** |  |
| Family | Intercept | 0.000 | 0.000 |  |
| Residual |  | 408644.000 | 639.300 |  |
|  |  |  |  |  |
| Fixed effects |  |  |  |  |
| **Source of variation** | **Estimate** | **s.e.** | **t** | **P** |
| Intercept | 4291.830 | 1156.990 | 3.709 | 0.002 |
| Diet quality | 419.870 | 591.480 | 0.710 | 0.488 |
| Signal line | 500.780 | 500.480 | 1.001 | 0.332 |
| Hindwing size | -20.400 | 17.500 | -1.166 | 0.261 |
| Diet * Signal line | -632.580 | 692.420 | -0.914 | 0.375 |
|  |  |  |  |  |
| **Brightness of black in male's hindwings** |  |  |  |  |
| (n = 24, families = 19) |  |  |  |  |
|  |  |  |  |  |
| Random effects |  |  |  |  |
| **Source of variation** | **Name** | **σ2** | **SD** |  |
| Family | Intercept | 281576.000 | 530.600 |  |
| Residual |  | 85350.000 | 292.100 |  |
|  |  |  |  |  |
| Fixed effects |  |  |  |  |
| **Source of variation** | **Estimate** | **s.e.** | **t** | **P** |
| Intercept | 4149.634 | 1053.835 | 3.938 | 0.007 |
| Diet quality | -53.741 | 409.763 | -0.131 | 0.897 |
| Signal line | 551.336 | 382.180 | 1.443 | 0.166 |
| Hindwing size | -10.177 | 14.920 | -0.682 | 0.523 |
| Diet * Signal line | -148.133 | 445.841 | -0.332 | 0.743 |
|  |  |  |  |  |
| **Saturation of colourful pigmentation in female's forewings** | |  |  |  |
| (n = 21, families = 15) |  |  |  |  |
|  |  |  |  |  |
| Random effects |  |  |  |  |
| **Source of variation** | **Name** | **σ2** | **SD** |  |
| Family | Intercept | 0.000 | 0.015 |  |
| Residual |  | 0.000 | 0.013 |  |
|  |  |  |  |  |
| Fixed effects |  |  |  |  |
| **Source of variation** | **Estimate** | **s.e.** | **t** | **P** |
| Intercept | 0.199 | 0.045 | 4.432 | 0.002 |
| Diet quality | -0.018 | 0.018 | -1.020 | 0.327 |
| Signal line | -0.003 | 0.016 | -0.221 | 0.829 |
| Forewing size | 0.001 | 0.000 | 1.531 | 0.169 |
| Diet * Signal line | 0.006 | 0.020 | 0.318 | 0.755 |
|  |  |  |  |  |
| **Saturation of colourful pigmentation in male's forewings** | |  |  |  |
| (n = 24, families = 19) |  |  |  |  |
|  |  |  |  |  |
| Random effects |  |  |  |  |
| **Source of variation** | **Name** | **σ2** | **SD** |  |
| Family | Intercept | 0.000 | 0.000 |  |
| Residual |  | 0.001 | 0.028 |  |
|  |  |  |  |  |
| Fixed effects |  |  |  |  |
| **Source of variation** | **Estimate** | **s.e.** | **t** | **P** |
| Intercept | 0.349 | 0.052 | 6.723 | <0.001 |
| Diet quality | -0.017 | 0.019 | -0.894 | 0.382 |
| Signal line | -0.024 | 0.019 | -1.248 | 0.227 |
| Forewing size | -0.001 | 0.000 | -1.972 | 0.063 |
| Diet * Signal line | 0.017 | 0.024 | 0.708 | 0.488 |
|  |  |  |  |  |
| **Saturation of colourful patterns in female's hindwings** | |  |  |  |
| (n = 21, families = 15) |  |  |  |  |
|  |  |  |  |  |
| Random effects |  |  |  |  |
| **Source of variation** | **Name** | **σ2** | **SD** |  |
| Family | Intercept | 0.000 | 0.000 |  |
| Residual |  | 0.001 | 0.029 |  |
|  |  |  |  |  |
| Fixed effects |  |  |  |  |
| **Source of variation** | **Estimate** | **s.e.** | **t** | **P** |
| Intercept | 0.334 | 0.053 | 6.295 | <0.001 |
| Diet quality | -0.022 | 0.027 | -0.812 | 0.429 |
| Signal line | 0.000 | 0.023 | 0.016 | 0.987 |
| Hindwing size | 0.000 | 0.001 | -0.291 | 0.775 |
| Diet * Signal line | 0.017 | 0.032 | 0.521 | 0.610 |
|  |  |  |  |  |
| **Saturation of colourful patterns in male's hindwings** | |  |  |  |
| (n = 24, families = 19) |  |  |  |  |
|  |  |  |  |  |
| Random effects |  |  |  |  |
| **Source of variation** | **Name** | **σ2** | **SD** |  |
| Family | Intercept | 0.002 | 0.041 |  |
| Residual |  | 0.004 | 0.064 |  |
|  |  |  |  |  |
| Fixed effects |  |  |  |  |
| **Source of variation** | **Estimate** | **s.e.** | **t** | **P** |
| Intercept | 0.841 | 0.168 | 4.991 | 0.000 |
| Diet quality | 0.014 | 0.052 | 0.269 | 0.791 |
| Signal line | 0.039 | 0.051 | 0.759 | 0.457 |
| Hindwing size | -0.009 | 0.002 | -3.721 | 0.002 |
| Diet * Signal line | -0.024 | 0.063 | -0.378 | 0.710 |
|  |  |  |  |  |
| **Saturation of black in female's forewings** |  |  |  |  |
| (n = 21, families = 15) |  |  |  |  |
|  |  |  |  |  |
| Random effects |  |  |  |  |
| **Source of variation** | **Name** | **σ2** | **SD** |  |
| Family | Intercept | <0.001 | 0.008 |  |
| Residual |  | <0.001 | 0.015 |  |
|  |  |  |  |  |
| Fixed effects |  |  |  |  |
| **Source of variation** | **Estimate** | **s.e.** | **t** | **P** |
| Intercept | -0.021 | 0.044 | -0.483 | 0.636 |
| Diet quality | -0.011 | 0.015 | -0.685 | 0.503 |
| Signal line | 0.017 | 0.014 | 1.228 | 0.237 |
| Forewing size | 0.001 | 0.000 | 2.031 | 0.061 |
| Diet * Signal line | 0.012 | 0.017 | 0.684 | 0.504 |
|  |  |  |  |  |
| **Saturation of black in male's forewings** |  |  |  |  |
| (n = 24, families = 19) |  |  |  |  |
|  |  |  |  |  |
| Random effects |  |  |  |  |
| **Source of variation** | **Name** | **σ2** | **SD** |  |
| Family | Intercept | <0.001 | 0.014 |  |
| Residual |  | <0.001 | 0.011 |  |
|  |  |  |  |  |
| Fixed effects |  |  |  |  |
| **Source of variation** | **Estimate** | **s.e.** | **t** | **P** |
| Intercept | 0.128 | 0.032 | 3.956 | 0.001 |
| Diet quality | 0.015 | 0.012 | 1.280 | 0.217 |
| Signal line | 0.010 | 0.011 | 0.831 | 0.416 |
| Forewing size | -0.001 | 0.000 | -2.018 | 0.059 |
| Diet * Signal line | -0.010 | 0.014 | -0.733 | 0.473 |
|  |  |  |  |  |
| **Saturation of black in female's hindwings** |  |  |  |  |
| (n = 21, families = 15) |  |  |  |  |
|  |  |  |  |  |
| Random effects |  |  |  |  |
| **Source of variation** | **Name** | **σ2** | **SD** |  |
| Family | Intercept | 0.000 | 0.000 |  |
| Residual |  | <0.001 | 0.016 |  |
|  |  |  |  |  |
| Fixed effects |  |  |  |  |
| **Source of variation** | **Estimate** | **s.e.** | **t** | **P** |
| Intercept | 0.041 | 0.029 | 1.391 | 0.183 |
| Diet quality | -0.011 | 0.015 | -0.721 | 0.481 |
| Signal line | 0.018 | 0.013 | 1.381 | 0.186 |
| Hindwing size | 0.000 | 0.000 | 0.338 | 0.740 |
| Diet * Signal line | -0.005 | 0.018 | -0.270 | 0.791 |
|  |  |  |  |  |
| **Saturation of black in male's hindwings** |  |  |  |  |
| (n = 24, families = 19) |  |  |  |  |
|  |  |  |  |  |
| Random effects |  |  |  |  |
| **Source of variation** | **Name** | **σ2** | **SD** |  |
| Family | Intercept | <0.001 | 0.024 |  |
| Residual |  | <0.001 | 0.010 |  |
|  |  |  |  |  |
| Fixed effects |  |  |  |  |
| **Source of variation** | **Estimate** | **s.e.** | **t** | **P** |
| Intercept | 0.105 | 0.039 | 2.666 | 0.035 |
| Diet quality | 0.006 | 0.017 | 0.329 | 0.746 |
| Signal line | -0.003 | 0.016 | -0.187 | 0.854 |
| Hindwing size | -0.001 | 0.001 | -1.139 | 0.304 |
| Diet * Signal line | 0.004 | 0.018 | 0.243 | 0.811 |
|  |  |  |  |  |
| **Hindwing size in females** |  |  |  |  |
| (n = 53, families = 30) |  |  |  |  |
|  |  |  |  |  |
| Random effects |  |  |  |  |
| **Source of variation** | **Name** | **σ2** | **SD** |  |
| Family | Intercept | 0.000 | 0.000 |  |
| Residual |  | 129.600 | 11.380 |  |
|  |  |  |  |  |
| Fixed effects |  |  |  |  |
| **Source of variation** | **Estimate** | **s.e.** | **t** | **P** |
| Intercept | 58.514 | 4.025 | 14.537 | <0.001 |
| Diet quality | 10.574 | 5.532 | 1.911 | 0.062 |
| Signal line | 9.893 | 4.838 | 2.045 | 0.046 |
| Diet * Signal line | -15.520 | 6.709 | -2.313 | 0.025 |
|  |  |  |  |  |
| **Hindwing size in males** |  |  |  |  |
| (n = 53, families = 27) |  |  |  |  |
|  |  |  |  |  |
| Random effects |  |  |  |  |
| **Source of variation** | **Name** | **σ2** | **SD** |  |
| Family | Intercept | 34.230 | 5.851 |  |
| Residual |  | 83.760 | 9.152 |  |
|  |  |  |  |  |
| Fixed effects |  |  |  |  |
| **Source of variation** | **Estimate** | **s.e.** | **t** | **P** |
| Intercept | 73.642 | 3.633 | 20.268 | <0.001 |
| Diet quality | -3.299 | 4.624 | -0.713 | 0.479 |
| Signal line | -1.102 | 5.269 | -0.209 | 0.835 |
| Diet * Signal line | 5.360 | 6.182 | 0.867 | 0.390 |
|  |  |  |  |  |
| **Forewing size in females** |  |  |  |  |
| (n = 54, families = 31) |  |  |  |  |
|  |  |  |  |  |
| Random effects |  |  |  |  |
| **Source of variation** | **Name** | **σ2** | **SD** |  |
| Family | Intercept | 0.000 | 0.000 |  |
| Residual |  | 155.800 | 12.480 |  |
|  |  |  |  |  |
| Fixed effects |  |  |  |  |
| **Source of variation** | **Estimate** | **s.e.** | **t** | **P** |
| Intercept | 94.740 | 4.161 | 22.769 | <0.001 |
| Diet quality | 3.978 | 5.884 | 0.676 | 0.502 |
| Signal line | 7.788 | 5.096 | 1.528 | 0.133 |
| Diet * Signal line | -5.023 | 7.207 | -0.697 | 0.489 |
|  |  |  |  |  |
| **Forewing size in males** |  |  |  |  |
| (n = 54, families = 27) |  |  |  |  |
|  |  |  |  |  |
| Random effects |  |  |  |  |
| **Source of variation** | **Name** | **σ2** | **SD** |  |
| Family | Intercept | 22.230 | 4.715 |  |
| Residual |  | 167.600 | 12.946 |  |
|  |  |  |  |  |
| Fixed effects |  |  |  |  |
| **Source of variation** | **Estimate** | **s.e.** | **t** | **P** |
| Intercept | 103.506 | 4.343 | 23.835 | <0.001 |
| Diet quality | 3.775 | 5.919 | 0.638 | 0.527 |
| Signal line | 14.216 | 6.549 | 2.171 | 0.0355 * |
| Diet * Signal line | -7.893 | 8.087 | -0.976 | 0.334 |

Table S3. The effect of resource content of the diet and signal selection line on brightness of adult coloration.

| **Source of variation** | |  | **NumDF** | **DenDF** | **F** | **P** |
| --- | --- | --- | --- | --- | --- | --- |
| **Brightness of colourful patterns in forewings in females** | | | | | |  |
| Diet |  |  | 1 | 12.528 | 1.488 | 0.245 |
| Signal line |  |  | 1 | 12.528 | 0.005 | 0.944 |
| Diet x Signal line | |  | 1 | 12.528 | 0.071 | 0.794 |
| forewing size | |  | 1 | 12.528 | 5.449 | 0.037* |
|  |  |  |  |  |  |  |
| **Brightness of black patterns in forewings in females** | | | | | |  |
| Diet |  |  | 1 | 12.5 | 1.007 | 0.335 |
| Signal line |  |  | 1 | 9 | 0.1 | 0.759 |
| Diet x Signal line | |  | 1 | 12.35 | 0.0002 | 0.988 |
| forewing size | |  | 1 | 3.1 | 8.754 | 0.057 |
|  |  |  |  |  |  |  |
| **Brightness of black patterns in forewings in males** | | | | | |  |
| Diet |  |  | 1 | 13.8 | 0.838 | 0.376 |
| Signal line |  |  | 1 | 13.9 | 0.799 | 0.387 |
| Diet x Signal line | |  | 1 | 12.6 | 0.15 | 0.705 |
| forewing size | |  | 1 | 14 | 0.207 | 0.656 |
|  |  |  |  |  |  |  |
| **Brightness of colourful patterns in hindwings in females** | | | | |  |  |
| Diet |  |  | 1 | 14.9 | 0.017 | 0.898 |
| Signal line |  |  | 1 | 12.6 | 1.3 | 0.276 |
| Diet x Signal line | |  | 1 | 15.2 | 1.367 | 0.260 |
| forewing size | |  | 1 | 8.2 | 7.892 | 0.022* |
|  |  |  |  |  |  |  |
| **Brightness of colourful patterns in hindwings in males** | | | | |  |  |
| Diet |  |  | 1 | 69.1 | 0.0002 | 0.988 |
| Signal line |  |  | 1 | 69.1 | 0.658 | 0.420 |
| Diet x Signal line | |  | 1 | 69.1 | 0.877 | 0.352 |
| forewing size | |  | 1 | 69.1 | 12.074 | <0.001* |
|  |  |  |  |  |  |  |
| **Brightness of black patterns in hindwings in females** | | | | | |  |
| Diet |  |  | 1 | 16.8 | 0.097 | 0.760 |
| Signal line |  |  | 1 | 16.8 | 0.295 | 0.594 |
| Diet x Signal line | |  | 1 | 16.8 | 0.835 | 0.374 |
| forewing size | |  | 1 | 16.8 | 1.36 | 0.260 |
|  |  |  |  |  |  |  |
| **Brightness of black patterns in hindwings in males** | | | | | |  |
| Diet |  |  | 1 | 16.8 | 0.097 | 0.760 |
| Signal line |  |  | 1 | 16.8 | 0.295 | 0.594 |
| Diet x Signal line | |  | 1 | 16.8 | 1.36 | 0.374 |
| forewing size | |  | **1** | 16.8 | 0.835 | 0.260 |
|  |  |  |  |  |  |  |

* The brightness of colourful forewing pigmentation in males was analysed with Type III Wald’s Chi Square test due to errors in calculation of the Satterthwaite’s approximation. Diet, signal line or their interactions did not affect significantly the brightness of the pigmentation in light pattern elements in males (all P-values > 0.274). Hindwing size had a marginally non-significant effect on pigmentation ( X^2^= 3.674, df = 1, P = 0.06).

Table S4. The effect of resource content of the diet and signal selection line on saturation of adult coloration.

| **Source of variation** | | | **NumDF** | **DenDF** | **F** | **P** |
| --- | --- | --- | --- | --- | --- | --- |
| **Saturation of colourful patterns in forewings in females** | | | | | |  |
| Diet |  |  | 1 | 15.1 | 2.359 | 0.145 |
| Signal line | |  | 1 | 11.6 | <0.001 | 0.975 |
| Diet x Signal line | |  | 1 | 14.9 | 0.101 | 0.755 |
| forewing size | |  | 1 | 7.2 | 2.343 | 0.169 |
|  |  |  |  |  |  |  |
| **Saturation of colourful patterns in forewings in males** | | | | | |  |
| Diet |  |  | 1 | 19 | 0.459 | 0.506 |
| Signal line | |  | 1 | 19 | 1.532 | 0.231 |
| Diet x Signal line | |  | 1 | 19 | 0.501 | 0.488 |
| forewing size | |  | 1 | 19 | 3.888 | 0.063 |
|  |  |  |  |  |  |  |
| **Saturation of black patterns in forewings in females** | | | | | |  |
| Diet |  |  | 1 | 16 | 0.279 | 0.605 |
| Signal line | |  | 1 | 15.3 | 5.987 | 0.027* |
| Diet x Signal line | |  | 1 | 16 | 0.469 | 0.504 |
| forewing size | |  | 1 | 14.7 | 4.124 | 0.061 |
|  |  |  |  |  |  |  |
| **Saturation of black patterns in forewings in males** | | | | | |  |
| Diet |  |  | 1 | 18.8 | 2.240 | 0.151 |
| Signal line | |  | 1 | 17.4 | 0.310 | 0.584 |
| Diet x Signal line | |  | 1 | 18.6 | 0.538 | 0.473 |
| forewing size | |  | 1 | 18.2 | 4.070 | 0.059 |
|  |  |  |  |  |  |  |
| **Saturation of colourful patterns in hindwings in females** | | | | |  |  |
| Diet |  |  | 1 | 16 | 0.811 | 0.381 |
| Signal line | |  | 1 | 16 | 0.307 | 0.587 |
| Diet x Signal line | |  | 1 | 16 | 0.271 | 0.610 |
| forewing size | |  | 1 | 16 | 0.084 | 0.775 |
|  |  |  |  |  |  |  |
| **Saturation of colourful patterns in hindwings in males** | | | | |  |  |
| Diet |  |  | 1 | 17.1 | 0.004 | 0.951 |
| Signal line | |  | 1 | 16.8 | 0.616 | 0.444 |
| Diet x Signal line | |  | 1 | 16.7 | 0.143 | 0.710 |
| forewing size | |  | 1 | 17.4 | 13.849 | 0.002* |
|  |  |  |  |  |  |  |
| **Saturation of black patterns in hindwings in females** | | | | | |  |
| Diet |  |  | 1 | 16 | 2.435 | 0.138 |
| Signal line | |  | 1 | 16 | 3.095 | 0.098 |
| Diet x Signal line | |  | 1 | 16 | 0.073 | 0.791 |
| forewing size | |  | 1 | 16 | 0.114 | 0.740 |
|  |  |  |  |  |  |  |
| **Saturation of black patterns in hindwings in males** | | | | | |  |
| Diet |  |  | 1 | 18.8 | 0.736 | 0.402 |
| Signal line | |  | 1 | 16.1 | 0.004 | 0.951 |
| Diet x Signal line | |  | 1 | 18.8 | 0.059 | 0.811 |
| forewing size | |  | 1 | 5.2 | 1.298 | 0.304 |
|  |  |  |  |  |  |  |
